# Deltex-3-like (DTX3L) stimulates metastasis of melanoma through FAK/PI3K/AKT but not MEK/ERK pathway

**DOI:** 10.18632/oncotarget.3742

**Published:** 2015-04-20

**Authors:** Nguyen Dinh Thang, Ichiro Yajima, Mayuko Y. Kumasaka, Machiko Iida, Tamio Suzuki, Masashi Kato

**Affiliations:** ^1^ Unit of Environmental Health Sciences, Department of Biomedical Sciences, College of Life and Health Sciences, Chubu University, Kasugai-shi, Aichi, Japan; ^2^ Department of Biochemistry and Plant Physiology, VNU University of Science, Vietnam National University, Hanoi, Vietnam; ^3^ Department of Occupational and Environmental Health, Nagoya University Graduate School of Medicine, Nagoya, Aichi, Japan; ^4^ Department of Dermatology, Yamagata University Faculty of Medicine, Yamagata, Japan

**Keywords:** Deltex-3-like, melanoma, metastasis, cancer, FAK/PI3K/AKT pathway

## Abstract

Deltex-3-like (DTX3L), an E3 ligase, is a member of the Deltex (DTX) family and is also called B-lymphoma and BAL-associated protein (BBAP). Previously, we established RFP/RET-transgenic mice, in which systemic hyperpigmented skin, benign melanocytic tumor(s) and melanoma(s) develop stepwise. Here we showed that levels of Dtx3l/DTX3L in spontaneous melanoma in RFP/RET-transgenic mice and human melanoma cell lines were significantly higher than those in benign melanocytic cells and primarily cultured normal human epithelial melanocytes, respectively. Immunohistochemical analysis of human tissues showed that more than 80% of the melanomas highly expressed DTX3L. Activity of FAK/PI3K/AKT signaling, but not that of MEK/ERK signaling, was decreased in Dtx3l/DTX3L-depleted murine and human melanoma cells. In summary, we demonstrated not only increased DTX3L level in melanoma cells but also DTX3L-mediated regulation of invasion and metastasis in melanoma through FAK/PI3K/AKT but not MEK/ERK signaling. Our analysis in human BRAF^V600E^ inhibitor-resistant melanoma cells showed about 80% decreased invasion in the DTX3L-depleted cells compared to that in the DTX3L-intact cells. Thus, DTX3L is clinically a potential therapeutic target as well as a potential biomarker for melanoma.

## INTRODUCTION

Previous studies showed that the incidence of melanoma, which is known as an aggressive cancer with high metastatic ability, is increasing at a greater rate than that of any other cancer [1, 2]. Melanoma accounts for less than 5% of all cutaneous carcinomas but is responsible for 80% of cutaneous cancer deaths [3]. Therefore, control of metastasis might be an important therapeutic target for melanoma.

Metastatic dissemination of a primary tumor to a secondary site is the major cause of deaths from solid tumors [4, 5]. The progression to metastasis involves a series of discrete steps, commonly known as the metastatic cascade. Tumor cells must invade from the primary tumor, dissociate from the tumor mass and be transported to nearby or distant secondary sites in the cascade [4]. Thus, cell invasion plays an essential role in the cascade. The cascade has been reported to be controlled by various signaling molecules such as BRAF/MEK/ERK [6, 7] and FAK/PI3K/AKT [8, 9]. The RAS/RAF/MEK/ERK pathway, one of the major pathways involved in melanoma progression, is regulated by receptor tyrosine kinases, cytokines and heterotrimeric G-protein-coupled receptors [6]. The small G protein RAS is localized to the plasma membrane and activates a downstream factor, RAF, followed by sequential activation of MEK and ERK [7]. Activation of BRAF/MEK/ERK signaling promotes invasion and metastasis of melanoma cells [10]. On the other hand, PI3K/AKT are potentially downstream of FAK in melanoma cells [8, 9]. Activation of FAK/PI3K/AKT signaling also promotes invasion and metastasis of melanoma cells [11, 12].

Deltex-3-like (DTX3L), an E3 ligase, is a member of the Deltex (DTX) family and is also called B-lymphoma and BAL-associated protein (BBAP). DTX3L was originally identified as a binding partner of B aggressive lymphoma 1 (BAL1), a risk-related gene and protein in diffuse large B cell lymphoma (DLBCL) [13, 14]. Expression of DTX3L transcript was detected in the thymus at the highest level [15]. Its expression was also detected in the telencephalic vesicles, hypothalamus, anterior pituitary, olfactory bulb, nasal cavity, mouth cavity, urogenital sinus, midgut loops and rectum [15]. Since DTX3L monoubiquitylates Histone H4 and selectively modulates the DNA damage response, lymphomas with increased expression level of DTX3L are resistant to DNA-damaging chemotherapeutic agents [16, 17]. Although a recent *in vitro* study showed the effect of DTX3L via STAT1 and IRF-1 in prostate cancer cells [18], *in vitro* studies on Dtx3l/DTX3L functions are limited to solid tumors, and *in vivo* studies are further limited.

Previously, we established *RFP/RET*-transgenic mice of line 304/B6 (RET-mice), in which systemic hyperpigmented skin, benign melanocytic tumor(s) and melanoma(s) develop stepwise [1]. RET-mice could be a powerful tool for analyzing the effects of molecules on melanomagenesis [1, 2]. Our previous DNA microarray analysis in a benign melanocytic tumor and a melanoma from a RET-mouse [19] showed increased levels of Dtx3l transcript in melanoma. The present *in vitro* and *in vivo* study newly clarified a function as well as expression level of DTX3L in murine and human melanomas.

## RESULTS

### Expression levels of Dtx3l in tumors from RET-mice and murine melanoma cell lines

**Figure 1:**
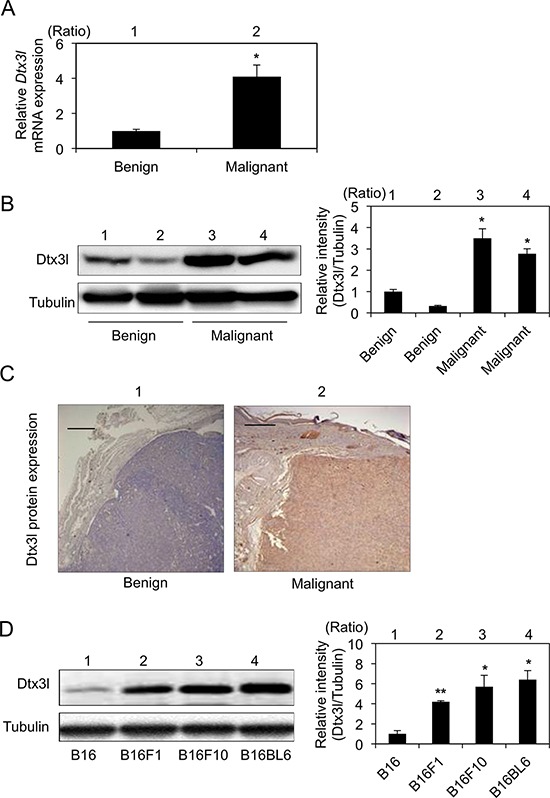
Increased expression levels of Dtx3l in melanoma tissues and cells in mice Expression levels (means ± SD) of *Dtx3*l transcript **A.** in benign melanocytic tumors (lane 1; *n* = 4) and melanomas (lane 2; *n* = 4) from RET-mice by real-time PCR analysis are presented. Representative results for Dtx3l protein expression in benign tumors (lanes 1 and 2 in B, lane 1 in C) and melanomas (lanes 3 and 4 in B, lane 2 in C) from RET-mice by immunoblot **B.** and immunohistochemical **C.** analyses are presented. Expression levels of Dtx3l (means ± SD) determined by densitometric analyses of the bands in 3 independent experiments are presented as graphs showing relative intensities (lanes 2–4 in B) for a benign tumor (lane 1 in B). Expression levels of Dtx3l protein **D.** in B16 (lane 1), B16F1 (lane 2), B16F10 (lane 3) and B16BL6 (lane 4) melanoma cells determined by immunoblot analysis are presented. Expression levels of Dtx3l (means ± SD) determined by densitometric analyses of the bands in 3 independent experiments are presented as graphs showing relative intensities (lanes 2–4 in D) for B16 (lane 1 in D). Expression levels of α-Tubulin are presented as an internal control (B, D). *, Significantly different (*, *p* < 0.05; **, *p* < 0.01) by Dunnett's test. Scale bar, 200 μM.

After selection of Dtx3l from our previous results of DNA microarray analysis [20], we first examined expression levels of Dtx3l transcript and protein by real-time PCR (Figure 1A), immunoblot (Figure 1B) and immunohistochemical (Figure 1C) analyses in tumors from RET-mice. Real-time PCR analysis of tumors in RET-mice showed that Dtx3l transcript levels in melanomas were about 4-fold higher than those in benign tumors (Figure 1A). Immunoblot and immunohistochemical analyses also showed that Dtx3l protein expression levels in melanomas were increased compared with those in benign melanocytic tumors from RET-mice (Figure 1B and 1C). Moreover, Dtx3l protein expression levels in B16F1, B16F10 and B16BL6 cells were higher than the level in B16 cells (Figure 1D).

### Expression levels of DTX3L transcript and protein in human melanoma cell lines

**Figure 2:**
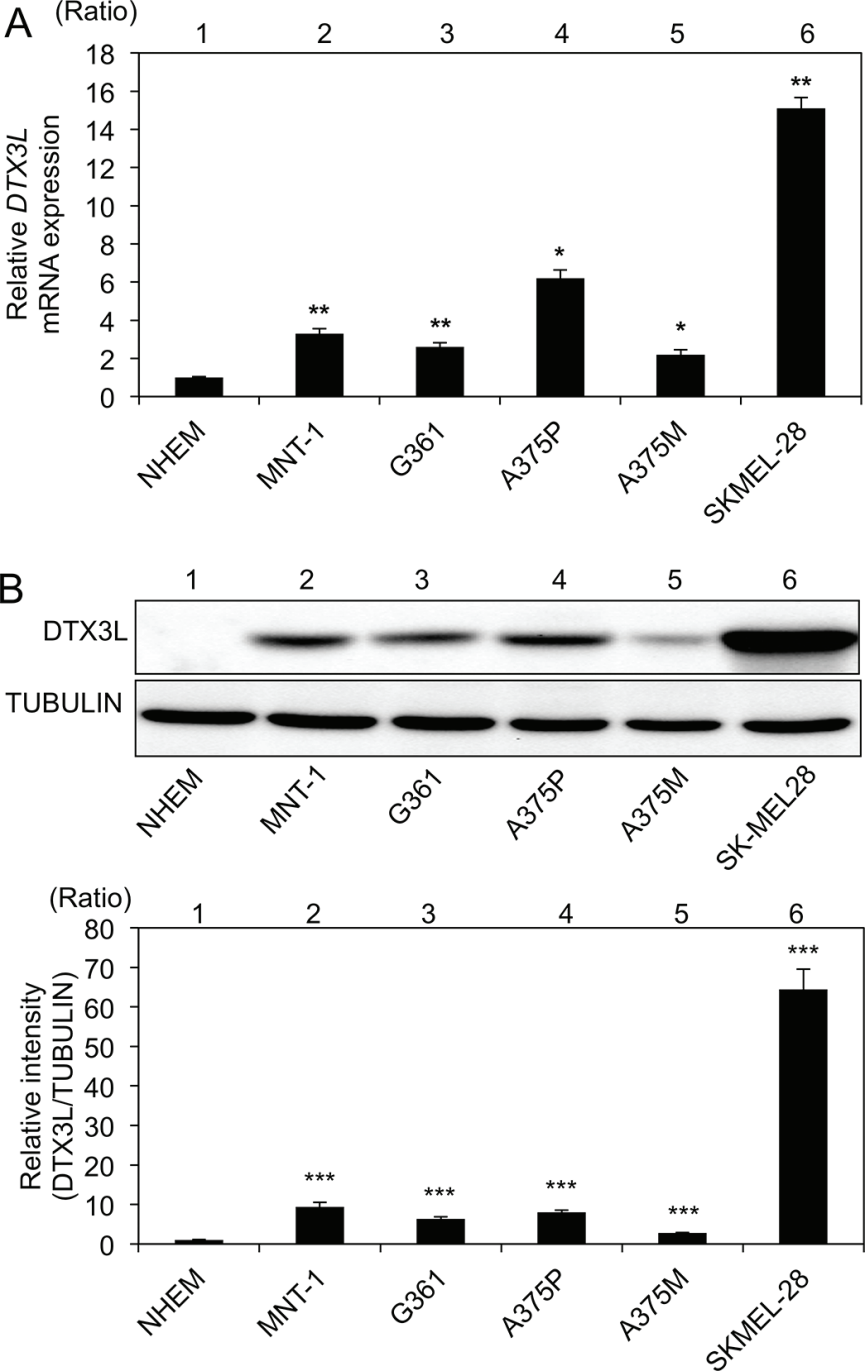
Increased expression levels of DTX3L in melanoma cells in humans Expression levels (means ± SD) of *DTX3L* transcript **A.** and protein **B.** in normal human epithelial melanocytes (NHEMs) and melanoma cells (MNT-1, G361, A375P, A375M and SK-Mel28) determined by real-time PCR A. and immunoblot B. analyses are presented. Expression levels of α-TUBULIN protein are presented as an internal control B. Expression levels of Dtx3l (means ± SD) determined by real-time PCR A. and densitometric analyses of the bands B. in 3 independent experiments are presented as graphs showing relative values (lanes 2–6) for NHEMs (lane 1). * and **, Significantly different (*, *p* < 0.05; **, *p* < 0.01) by Dunnett's test.

We next examined DTX3L transcript and protein expression levels in 6 human melanoma cell lines and NHEM cells. *DTX3L* transcript expression levels in all of the melanoma cell lines (MNT-1, G361, A375P, A375M and SK-Mel28) were significantly higher than the level in NHEM cells (Figure 2A). DTX3L protein expression levels in all of the cell lines were also higher than the level in NHEM cells (Figure 2B). These results showed that DTX3L transcript and protein expression levels are increased in human melanoma cell lines compared with those in normal human epithelial melanocytes.

### Expression levels of DTX3L protein in nevi and melanomas in humans

**Figure 3:**
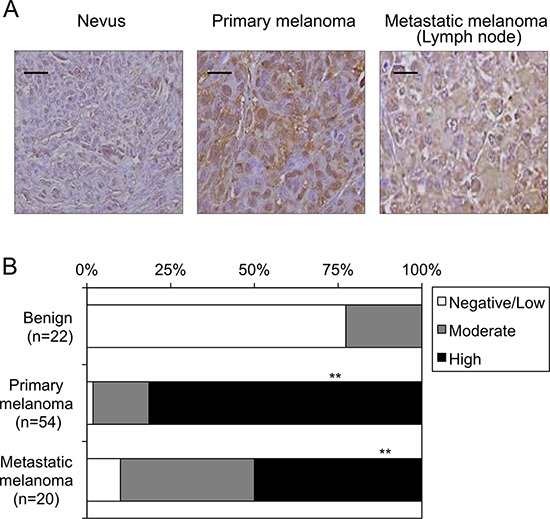
Expression levels of DTX3L in nevi and melanoma tissues in humans Representative photographs **A.** and results of statistical analysis **B.** of Dtx3l protein expression levels in nevi, primary melanomas and metastatic melanomas for lymph nodes in humans by immunohistochemical analysis are presented. White, gray and black columns show negative/low, moderate and high expression levels, respectively, of DTX3L protein expression levels in nevus, primary melanoma and metastatic melanoma tissues in humans B. Densitometric evaluation for the immunohistochemical results was performed using the software program WinROOF (MITANI Corporation) as previously reported (25). Number of DTX3L negatively/lowly, moderately and highly expressed cells was divided by number of total cells in five fields with 200-fold magnification in each tissue. Significantly different (**, *p* < 0.01) from nevi by Fisher's exact test. Scale bar, 25 μM.

DTX3L protein expression levels were immunohistochemically analyzed *in vivo* in human nevi (*n* = 22), primary melanomas (*n* = 54) and metastatic melanomas (*n* = 20) (Figure 3A and 3B). There were no nevi classified as high DTX3L expression (Figure 3B). Moreover, 77% of the nevi were classified as low or negative DTX3L expression (Figure 3B). In contrast, nevi classified as high or moderate expression of DTX3L were obtained in 98% of the primary melanomas and 90% of the metastatic melanomas (Figure 3B). Our results showed higher expression levels of DTX3L in melanomas than in nevi in humans.

### Decreased invasion in Dtx3l-depleted murine B16F10 melanoma cells

**Figure 4:**
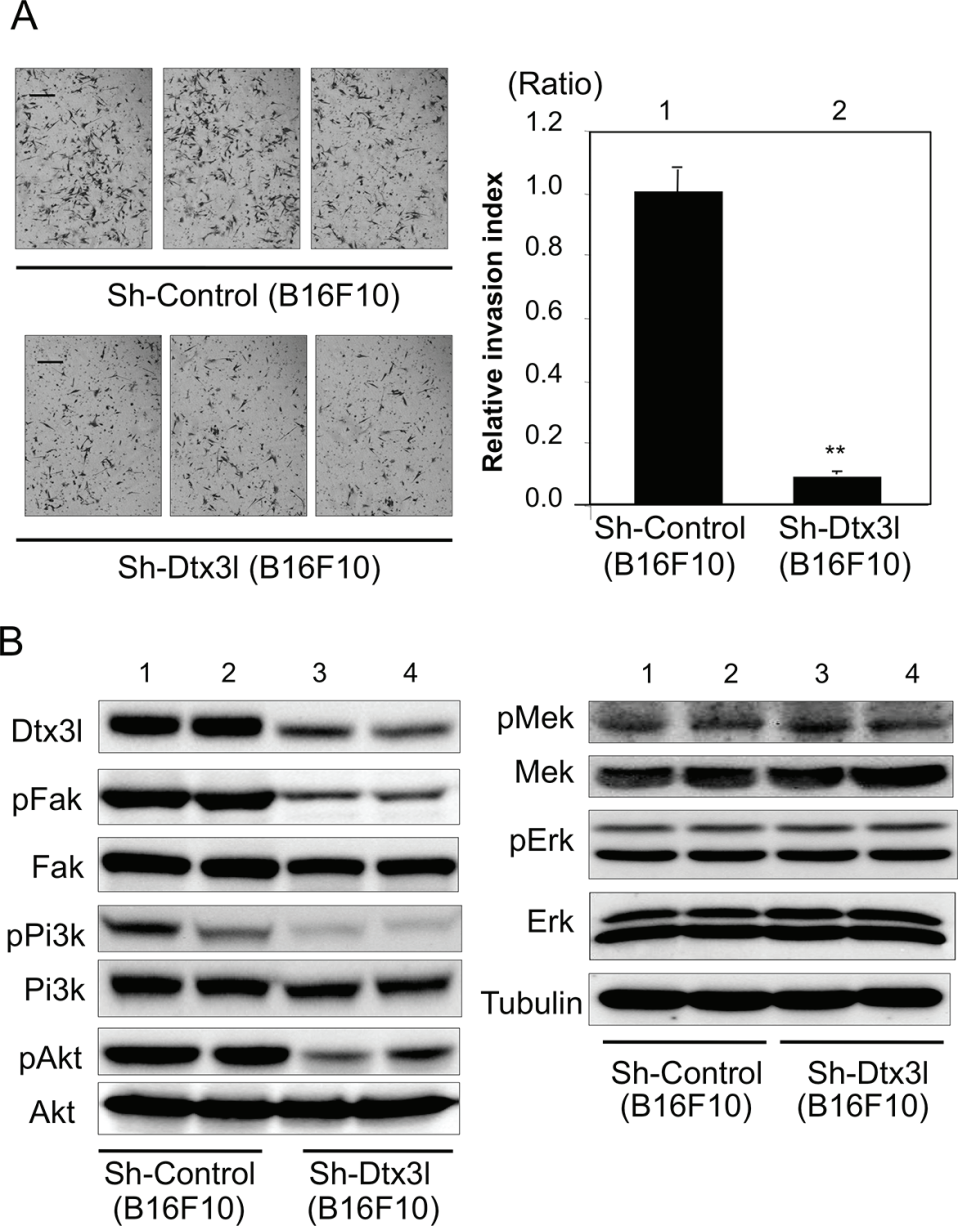
Decreased cell invasion of Dtx3l-depleted murine melanoma cells Matrigel-invasion assay was performed with control (Sh-Control) and Dtx3l-depleted (Sh-Dtx3l) B16F10 murine melanoma cells **A.** Photographs of cells invading the membrane stained with hematoxylin are presented (left). After invading cells had been counted in five random microscopic fields in each Matrigel-invasion assay, the results of 3 independent assays were normalized and are presented as an invasion index (right). Expression (Dtx3l, Fak, Pi3k, Akt, Mek and Erk) and phosphorylation (pFak, pPi3k, pAkt, pMek and pErk) levels in two kinds of DTX3L-depleted (Sh-DTX3L) and control (Sh-Control) murine melanoma cells determined by immunoblot analysis are presented **B.** Expression levels of α-Tubulin protein are presented as an internal control B. Significantly different (**, *p* < 0.01) from the control (Sh-Control) by Student's *t*-test. Scale bar, 50 μM.

We next tried to clarify the function of Dtx3l in murine B16F10 melanoma cells. Invasion activity (Figure 4A) and invasion-related signaling (Figure 4B) were examined after development of two stable control clones (lanes 1 and 2 in Figure 4B) and two stable Dtx3l-depleted clones (lanes 3 and 4 in Figure 4B). Invasion activity in Dtx3l-depleted B16F10 melanoma cells was less than 10% of that in control B16F10 melanoma cells. Phosphorylation levels of Fak, Pi3k and Akt in Dtx3l-depleted cells were decreased compared to those in control cells (Figure 4B). In addition, protein expression levels of Fak and Pi3k in Dtx3l-depleted cells were decreased compared to those in control cells, while Akt protein expression levels were comparable in Dtx3l-depleted and control cells. Phosphorylation levels of Mek and Erk in Dtx3l-depleted and control cells were comparable (Figure 4B).

### Decreased invasion in DTX3L-depleted human G361 melanoma cells

**Figure 5:**
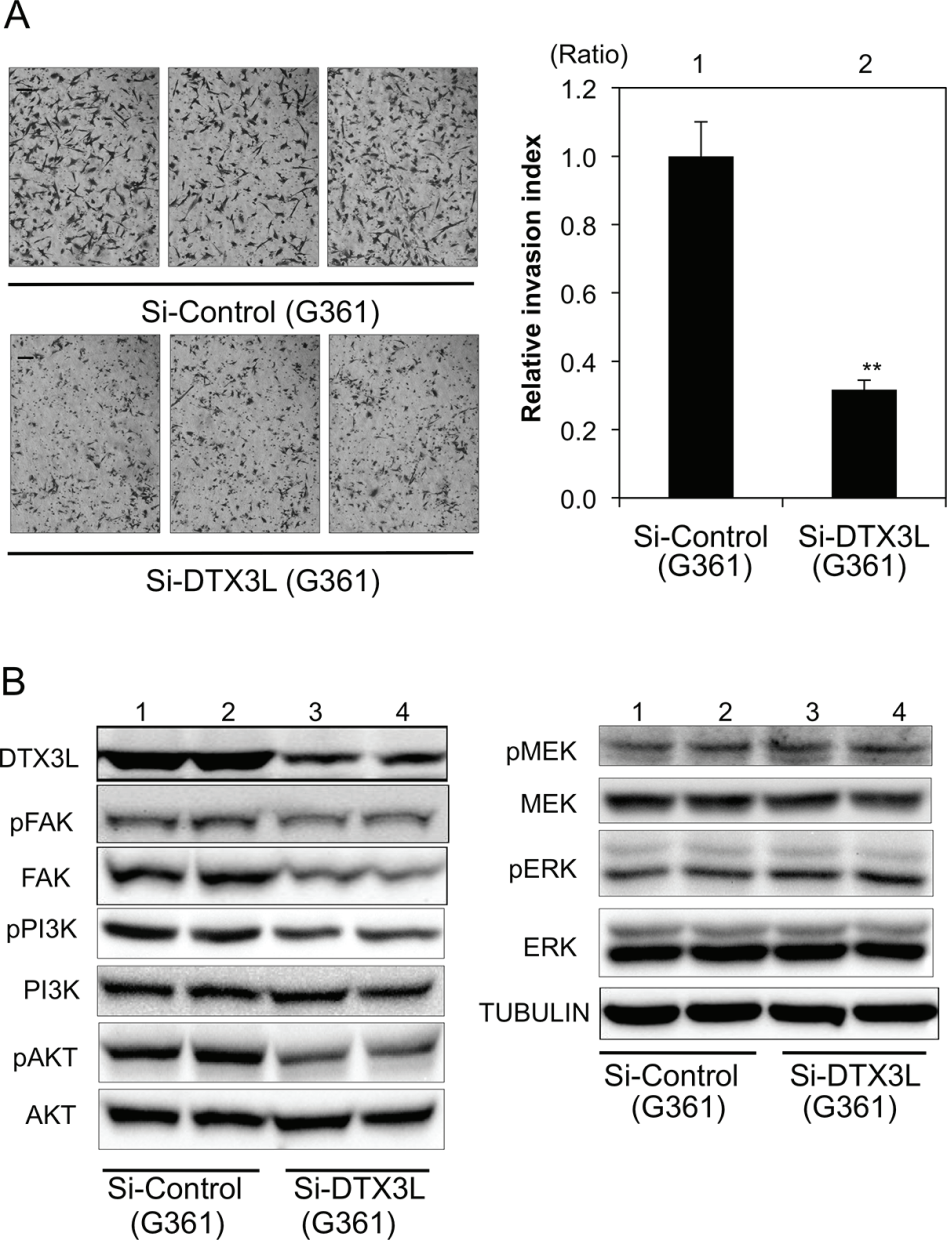
Decreased cell invasion of DTX3L-depleted human melanoma cells Matrigel-invasion assay was performed with control (Si-Control) and DTX3L-depleted (Si-DTX3L) G361 human melanoma cells **A.** Photographs of cells invading the membrane stained with hematoxylin are presented (left). After invading cells had been counted in five random microscopic fields in each Matrigel-invasion assay, the results of three independent assays were normalized and are presented as an invasion index (right). Expression (DTX3L, FAK, PI3K, AKT, MEK and ERK) and phosphorylation (pFAK, pPI3K, pAKT, pMEK and pERK) levels in two kinds of DTX3L-depeleted (Si-DTX3L) and control (Si-Control) human melanoma cells determined by immunoblot analysis are presented **B.** Expression levels of α-TUBULIN protein are presented as an internal control B. Significantly different (**, *p* < 0.01) from the control (Si-Control) by Student's *t*-test. Scale bar, 50 μM.

We next examined the function of DTX3L in human G361 melanoma cells. Invasion activity in DTX3L-depleted G361 melanoma cells was about 30% of that in control G361 melanoma cells. Corresponding to the murine melanoma cells, phosphorylation levels of FAK, PI3K and AKT in DTX3L-depleted cells were decreased compared to those in control cells (left Figure 5B). In addition, expression levels of FAK and PI3K in DTX3L-depleted cells were decreased compared to those in control cells, while AKT protein expression levels were comparable in DTX3L-depleted and control cells. Phosphorylation levels of MEK and ERK in DTX3L-depleted and control cells were comparable (right Figure 5B).

### Decreased invasion in Dtx3l-depleted murine B16F10 melanoma cells

**Figure 6:**
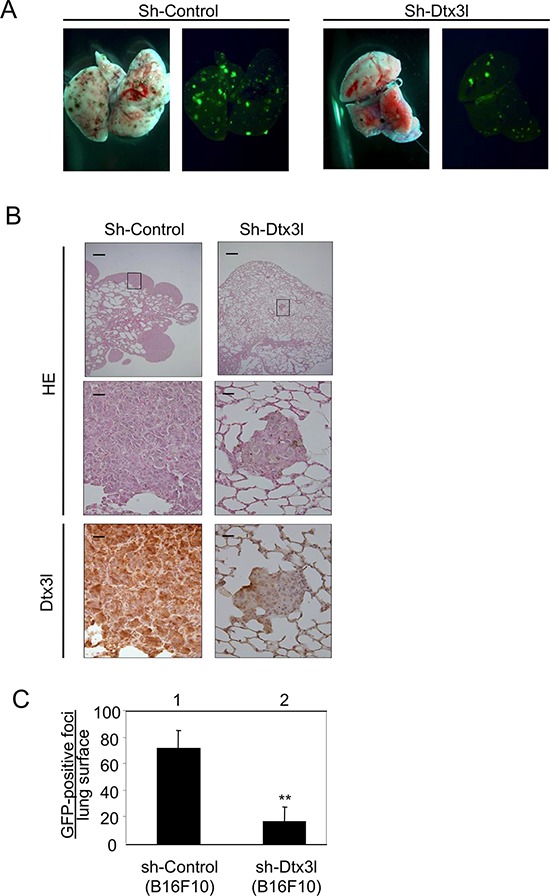
Decreased lung metastasis in Dtx3l-depleted melanoma cells *in vivo* Results of morphologic analysis of lung metastasis of control (Sh-Control) and Dtx3l-depleted (Sh-Dtx3l) murine B16F10 melanoma cells injected into tail veins of nude mice **A.** are presented. Animals were dissected to observe lung metastases at 14 days after inoculation. Lung metastases were macroscopically visualized by GFP fluorescence images. Metastatic foci derived from control (Sh-Control) and Dtx3l-depleted (Sh-Dtx3l) cells **B.** were microscopically confirmed by low (top panels in B) and high (middle and bottom panels in B) magnification of HE staining (HE) and immunohistochemistry (Dtx3l). Number of GFP-positive metastatic foci per lung surface **C.** after inoculation of control (Sh-Control; *n* = 4) and Dtx3l-depleted (Sh-Dtx3l; *n* = 4) murine B16F10 melanoma cells is presented. Significantly different (**, *p* < 0.01) from the control (Sh-Control) by the Student's *t*-test. Scale bar, 200 μM (low magnification) and 25 μM (high magnification).

Since previous studies showed that invasion activity is correlated with metastasis [21], we finally examined the effect of Dtx3l on metastasis *in vivo*. GFP-tagged Dtx3l-depleted B16F10 cells and control cells were injected into the tail veins of C57/BL6 mice. The number of metastatic foci in Dtx3l-depleted cells (Sh-Dtx3l) was reduced compared to that in control cells (Sh-Control) in our macroscopic analysis for fluorescence intensity on the surface of the lung (Figure 6A). Morphology and Dtx3l protein expression level in metastatic cells in the lung were confirmed by our microscopic analysis with HE staining and immunohistochemistry, respectively (Figure 6B). The number of GFP-positive metastatic foci on the surface of the lung derived from Dtx3l-depleted cells was about 20% of the number of foci derived from control cells in our statistical analysis (Figure 6C).

## DISCUSSION

To our knowledge, there has been no study showing functions of Dtx3l/DTX3L in solid tumors other than can *in vitro* study in prostate cancer cells [18]. Our *in vivo* study showed increased expression levels of Dtx3l in melanomas compared to the levels in murine benign melanocytic tumors in RET-mice. Our *in vitro* study showed demonstrated increased expression levels of DTX3L in melanoma cell lines compared to the level in NHEM cells in humans. More importantly, there was no nevus classified as high expression of DTX3L, while more than 80% of the melanomas were classified as high expression of DTX3L in our immunohistochemical analysis of human tissues. Our results suggest that the expression level of DTX3L protein, which is expressed in cytoplasmic areas of human melanoma cells (Supplementary Figure S1), is a potential biomarker for melanoma in humans.

We then tried to clarify the function of Dtx3l/DTX3L in melanoma. Our *in vitro* study showed 90% and 70% decreases in the invasion ability of Dtx3l/Dtx3l-depleted murine and melanoma cells, respectively, compared to that of control cells. In contrast, invasion ability *in vitro* was increased in DTX3L-overexpressed G361 melanoma cells (Supplementary Figure S2A). Moreover, our *in vivo* study showed more than 80% suppression of lung metastasis in Dtx3l-depleted melanoma cells. These results suggest that Dtx3l/DTX3L is a regulator of the invasion and metastasis for melanoma.

We finally tried to clarify the molecular mechanism of Dtx3l/DTX3L in melanoma. Cell invasion of the primary tumor, dissociation from the tumor mass, and transportation to nearby or distant secondary sites have been proposed as a process for metastasis of solid tumors [4]. Both FAK/PI3K/AKT and BRAF/MEK/ERK pathways have also been reported to regulate invasion and metastasis [6–8, 12, 22–24]. Regulators for PI3K/AKT and MEK/ERK pathways including c-Kit have also been suggested to be target molecules for melanoma prevention and therapy [22–25]. Our results obtained for murine and human melanoma cells showed that depletion of Dtx3l/DTX3L decreased the activity of Fak/FAK, Pi3k/PI3K and Akt/AKT. In contrast, overexpression of Dtx3l/DTX3L increased the activity of FAK and AKT (Supplementary Figure S2B). However, depletion of Dtx3l/DTX3L has a very limited effect on the activity of Mek/MEK and Erk/ERK in both murine and human melanoma cells. BRAF is a serine/threonine protein kinase that activates the MEK/ERK signaling pathway [26]. Previous studies revealed that approximately 50% of melanomas have activating BRAF mutations [26–28], and abundant data validate BRAF^V600E^ as a therapeutic target in melanoma [29–31]. Although drugs selectively inhibiting BRAF^V600E^ signaling could achieve dramatic clinical responses in melanoma patients with the *BRAF* mutation, most patients appear to eventually relapse [32]. Our results showed that Dtx3l/DTX3L-mediated regulation of melanoma metastasis is dependent on the FAK/PI3K/AKT pathway but not the MEK/ERK pathway. In fact, about 80% suppressed invasion activity (Supplementary Figure S3) was obtained in DTX3L-depleted human A375P melanoma cells with resistance to a specific inhibitor of BRAF^V600E^ [33]. Thus, DTX3L regulating the FAK/PI3K/AKT pathway is a potential target for melanoma patients who have relapsed after BRAF-targeted therapy.

In summary, our study suggested for the first time that Dtx3l/DTX3L is a potential therapeutic target as well as a potential biomarker for melanoma.

## METHODS

### Cells and mice

Normal human epithelial melanocyte (NHEM) cells (KURABO, Japan) were cultured in HMGS medium. Human melanoma cell lines of SK-Mel28 and G361 were obtained from Riken Bio Resource Center. A MNT1 cell line was a kind gift from Dr. VJ Hearing (National Cancer Institute, NIH, Bethesda, MD). Human melanoma cell lines of A375P and A375 and a murine nontumorigenic immortalized melanocyte (melan-a) cell line were kindly provided by Dr. Dorothy C Bennett, St George's, UK. Murine melanoma cell lines of B16, B16F1, B16F10 and B16BL6 were obtained from Cell Resource Center for Biomedical Research in Tohoku University. Benign melanocytic tumors and melanomas in transgenic mice of line 304/B6 (RET-mice) carrying constitutively activated RET [1, 2] were used.

### Real-time PCR

Total RNA was prepared from a frozen tumor sample and from murine and human cell line samples using a High Pure RNA Kit (Roche Diagnostics) according to the method previously described [34]. cDNA was then synthesized by reverse transcription of total RNA using Super-criptTMIII reverse transcriptase included in the RT enzyme mix and RT reaction mix according to the protocol previously described [34]. Real-time quantitative RT-PCR with SYBR green was performed using power SYBR1 Green PCR master mix (Applied Biosystems) in an ABI Prism7500 sequence detection system (Applied Biosystems). The expression levels of Dtx3l/DTX3L transcripts measured by quantitative RT-PCR (real-time PCR) were adjusted through the transcript expression level of hypoxanthine guanine phosphoribosyl transferase (Hprt) in mice and TATA-box-binding protein (TBP) in humans. PCR was carried out using 10 ml of power SYBR1 Green PCR master mix (Applied Biosystems) containing 900 nM forward primer and 900 nM reverse primer in a final volume of 20 ml. Sequences of primers for murine Hprt and human TBP and RET were shown in our previous report [19]. Sequences of primers for human DTX3L were 5′-AAA CAC CGT CTG GTG ATA TGC-3′ and 5′- GTA TGC CCT CTG CTC TTT GG-3′, and those for mouse Dtx3l were 5′-CGG GCT CGT TTC TAA CTC TG-3′ and 5′-CCA TCA CTA CCC TCC ATG CT-3′.

### Immunoblot and immunohistochemical analyses

Immunoblot and immunohistochemical analyses were performed according to the method described previously [18, 34]. Rabbit polyclonal antibodies against DTX3L/Dtx3l (Santa Cruz), phosphorylated threonine 202 in ERK1 and phosphorylated tyrosine 204 in ERK2 (Cell Signaling), phosphorylated tyrosine 397 in FAK (Invitrogen), phosphorylated MEK1/2 (Cell Signaling), PI3K and phosphorylated PI3K (Cell Signaling); rabbit monoclonal antibodies against Akt and phosphorylated Akt (Cell Signaling); and mouse monoclonal antibodies against alpha-TUBULIN (SIGMA), MEK1/2 (Cell Signaling), ERK1/2 (Cell Signaling) and FAK (Millipore) were used as first antibodies. Immunohistochemistry was performed according to the method previously described [34].

### Establishment of silencing and expression vectors of stable clones

Silencing vector pRNAT-U6–1-Neo (Invitrogen) was used for construction of the DTX3L silencing vector. A double-strand DNA fragment including a knockdown sequence for mouse Dtx3l was inserted into BamHI and HindIII sites. Control and DTX3L silencing vectors were transfected into B16F10 cells, and stable cell clones were selected with 1 mg/ml neomycin (Wako). Oligonucleotide sequences of the DNA fragment are 5′-GATC CGCATGGAGGGTAG TGATGGAATTAATTCA AGAGATTAATTCCATCACTACCCTCCATGCTTTTT TA-3′ and 5′- AGCTTAAAAAAGCATGGAGGGTAGTG ATGGAATTAATCTCTTGAATTAATTCCATCACTAC CCTCCATGCG-3′. Expression vector pCMV-c-Fa-Puro3 (Invitrogen) was used for construction of the DTX3L expression vector. The human DTX3L coding region fused with a FLAG sequence was inserted into BamHI and XhoI sites. Empty and DTX3L expression vectors were transfected into G361 cells, and stable cell clones were selected with 1 mg/ml puromycin (Wako).

### *In vitro* analysis of invasion and *in vivo* analysis of metastasis

Cell invasion ability was evaluated by an *in vitro* invasion assay according to the method previously reported [35], and *in vivo* analysis of metastasis was performed by the method previously reported [36]. After a stable clone of Dtx3l-depleted B16F10 cells (5 × 10^6^; *n* = 5) and control B16F10 cells (5 × 10^6^; *n* = 5) in 50 μl serum-free RPMI medium had been injected into tail veins of 6–8-week-old C57/BL6 mice, metastatic foci in the lung 14 days after inoculation were evaluated by fluorescence intensity.

### Permission

The Animal Care and Use Committee (approval no. 26317 in Nagoya University and 2410062 in Chubu University), the recombination DNA Advisory Committee (approval no. 13–76 in Nagoya University and 12–03 in Chubu University) and the ethical committee (approval number: 2013–0070 and 250007) in Nagoya University and Chubu University approved this study.

### Statistical analysis

Statistical analysis in this study was performed according to the method previously described [37]. Results from more than three independent experiments in each group were statistically analyzed by Dunnett's test, Fisher's exact test or Student's *t*-test.

## SUPPLEMENTARY FIGURES


