# Supplementary material for: Deltex-3-like (DTX3L) stimulates metastasis of melanoma through FAK/PI3K/AKT but not MEK/ERK pathway
Source: Oncotarget. 2015 Apr 20;6(16):14290–9. doi: 10.18632/oncotarget.3742 (PMC4546467; doi:10.18632/oncotarget.3742)
Supplement: Supplementary file 1 [file oncotarget-06-14290-s001.pdf]

SUPPLEMENTARY FIGURES

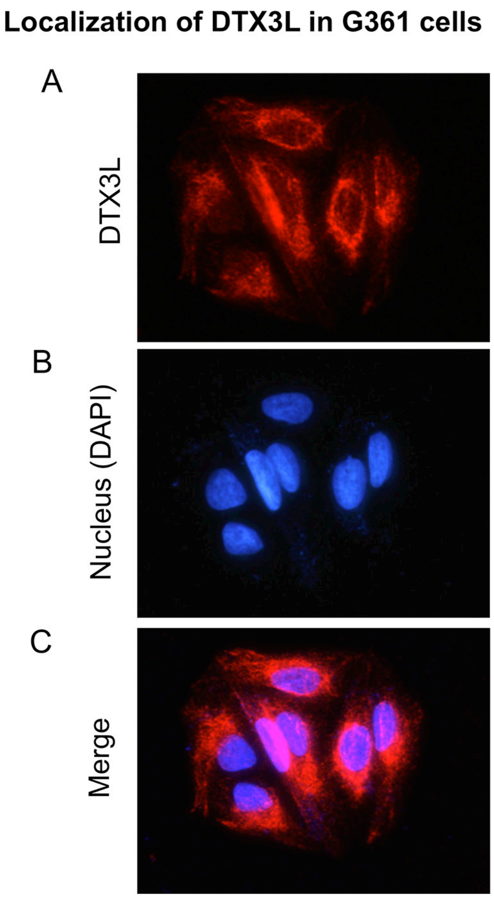

Supplementary Figure S1: Localization of DTX3L in human G361 melanoma cells. DTX3L protein expression was examined in G361 cells by immunocytochemistry with anti-DTX3L antibody (A and C) and DAPI counterstaining (B and C).

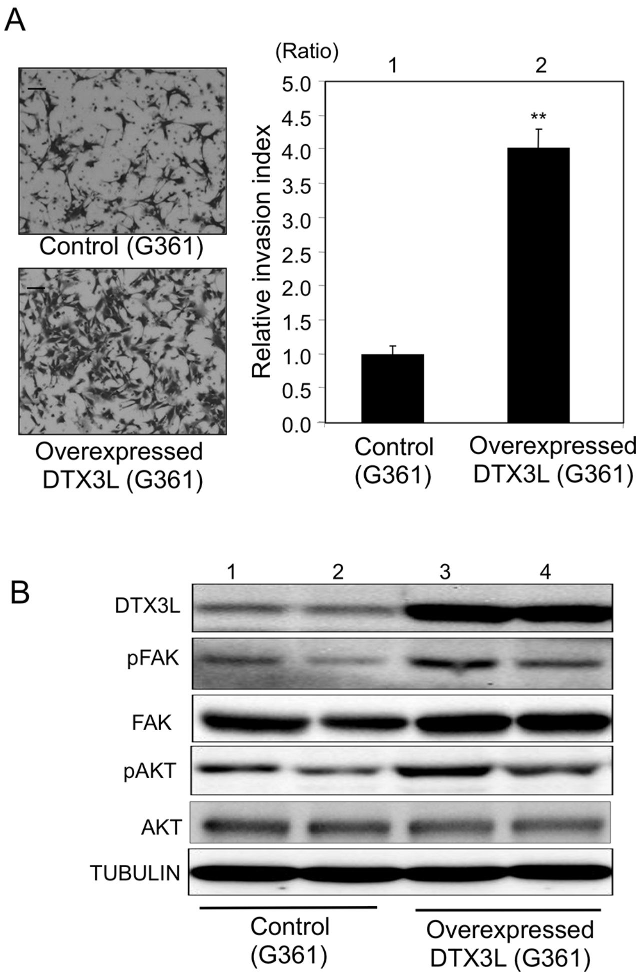

Supplementary Figure S2: Increased cell invasion of DTX3L-overexpressed human melanoma cells. Matrigel-invasion assay was performed with control and DTX3L-overexpressed G361 human melanoma cells **A.** Photographs of cells invading the membrane stained with hematoxylin are presented (left). After invading cells had been counted in five random microscopic fields in each Matrigel-invasion assay, the results of three independent assays were normalized and are presented as an invasion index (right). Expression (DTX3L, FAK and AKT) and phosphorylation (pFAK and pAKT) levels in control (lanes 1 and 2) and DTX3L-overexpressed (lanes 3 and 4) human melanoma cells are presented **B.** Expression levels of α-TUBULIN protein are presented as an internal control B. Significantly different (**, $p < 0.01$) from the control by Student's *t*-test. Scale bar, 50 μM.

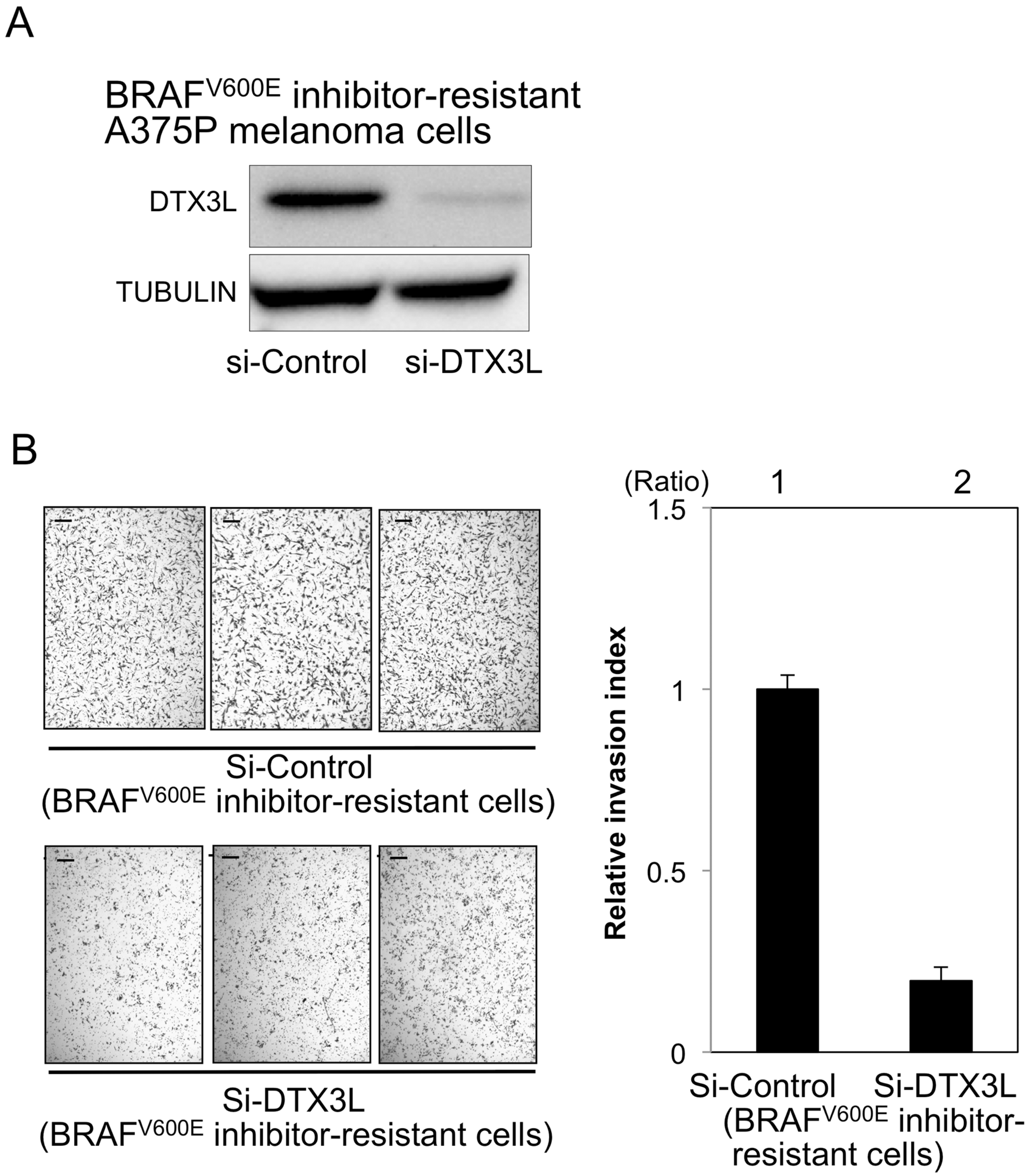

Supplementary Figure S3: Effect of decreased DTX3L expression level on invasion activity of BRAFV600E inhibitor-resistant A375 melanoma cells. A. Previously, human A375P melanoma cells with BRAF^{V600E} were cultured in RPMI (10% FBS) in the presence or absence of 5 μM of a specific inhibitor of BRAF^{V600E} for 3 months and BRAF^{V600E} inhibitor-resistant A375 melanoma cells were established (33). Matrigel-invasion assay was performed in control (Si-Control) and Dtx3l-depleted (Si-Dtx3l) BRAF^{V600E} inhibitor-resistant A375 cells **B.** Photographs of cells invading the membrane stained with hematoxylin are presented (left). After invading cells had been counted in five random microscopic fields in each Matrigel-invasion assay, the results of 3 independent assays were normalized and are presented as an invasion index (right). Significantly different (**, $p < 0.01$) from the control (Sh-Control) by Student's *t*-test. Scale bar, 50 μM.
